# Extracellular Vesicles in Glioblastoma Tumor Microenvironment

**DOI:** 10.3389/fimmu.2019.03137

**Published:** 2020-01-21

**Authors:** Anuroop Yekula, Anudeep Yekula, Koushik Muralidharan, Keiko Kang, Bob S. Carter, Leonora Balaj

**Affiliations:** ^1^Government General Hospital, Guntur Medical College, Guntur, India; ^2^Department of Neurosurgery, Massachusetts General Hospital and Harvard Medical School, Boston, MA, United States; ^3^School of Medicine, University of California, San Diego, La Jolla, CA, United States

**Keywords:** tumor microenvironment, glioblastoma, extracellular vesicles, immunomodulation, angiogenesis

## Abstract

Glioblastomas (GBM) are highly aggressive primary brain tumors. Complex and dynamic tumor microenvironment (TME) plays a crucial role in the sustained growth, proliferation, and invasion of GBM. Several means of intercellular communication have been documented between glioma cells and the TME, including growth factors, cytokines, chemokines as well as extracellular vesicles (EVs). EVs carry functional genomic and proteomic cargo from their parental cells and deliver that information to surrounding and distant recipient cells to modulate their behavior. EVs are emerging as crucial mediators of establishment and maintenance of the tumor by modulating the TME into a tumor promoting system. Herein we review recent literature in the context of GBM TME and the means by which EVs modulate tumor proliferation, reprogram metabolic activity, induce angiogenesis, escape immune surveillance, acquire drug resistance and undergo invasion. Understanding the multifaceted roles of EVs in the niche of GBM TME will provide invaluable insights into understanding the biology of GBM and provide functional insights into the dynamic EV-mediated intercellular communication during gliomagenesis, creating new opportunities for GBM diagnostics and therapeutics.

## Introduction

Glioblastomas are the most common malignant primary brain tumors in adults. They are highly aggressive and have an overall survival of <15 months despite maximal surgical resection and chemoradiation (1). GBM has several unique features that characterize its intrinsic aggressive behavior and unresponsiveness to the therapy. GBMs are typically heterogeneous with a wide range of genetic and epigenetic variations among tumor cells. Moreover, glioma cells reside in a niche of stromal cells and communicate with them to modify their functions to establish a tumor promoting environment. GBM TME consists of glioma cells, specialized glioma stem cells (GSC), stromal cells including resident glial cells (oligodendrocytes, astrocytes, ependymal cells, microglia), and infiltrating immune cells such as monocytes, macrophages and lymphocytes (2, 3).

**Figure 1:**
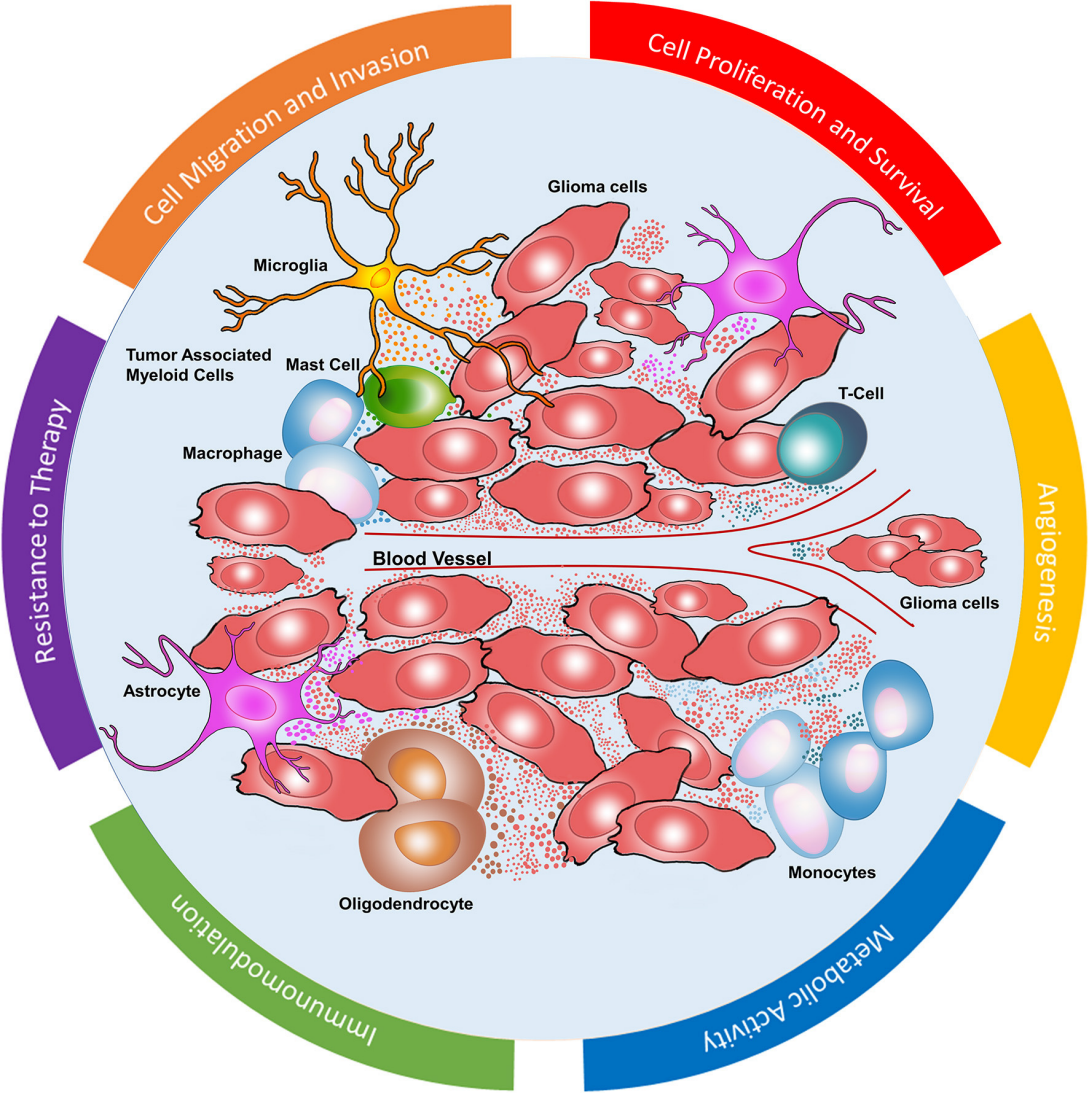
Glioblastoma microenvironment. Dynamic EV mediated communication between glioma cells and stromal cells including monocytes, macrophages, mast cells, microglia, T cells, astrocytes, and oligodendrocytes. EVs in GBM microenvironment mediate cell proliferation and survival, angiogenesis, metabolic activity, immunomodulation, resistance to chemoradiation as well as cell migration and invasion.

Tumor cells develop a symbiotic relationship with different stromal cells to shift tissue homeostasis toward a tumor supporting microenvironment by dynamically communicating with stromal cells bi-directionally via cell-cell transversing gap junctions, tunneling nanotubes and secretion of effector molecules including growth factors, cytokines, chemokines and extracellular vesicles (EVs) (4). EVs are membrane-bound submicron vesicles released by all cells into their microenvironment which are implicated in intercellular communication in both physiological and pathological conditions. EVs carry functional genomic and proteomic cargo from their parental cells and deliver that information to surrounding and distant recipient cells to modulate their behavior. EVs include a broad range of vesicles including exosomes (50–200 nm), microvesicles (>100–1 μm), apoptotic bodies (50–2,000 nm) and large oncosomes(>1 μm) (4, 5). Recent studies have highlighted the multifaceted role of EVs in supporting several hallmarks of cancer such as tumor proliferation, evasion of cell death, inducing angiogenesis, modifying metabolism, invasion, and metastasis (6). Here, we elaborate the dynamic role of EVs in modulating the GBM TME into a tumor supporting system (Figure 1). This knowledge is invaluable in understanding the complex heterogeneous biology of GBM and gain functional insights into the dynamic EV mediated intercellular communication in gliomagenesis (3, 7). Understanding EV mediated modulations of TME can unveil novel diagnostic and therapeutic targets. Navigating through TME, this review will provide functional insights that EVs bear in several aspects of GBM progression.

## Glioma EVs Enhance Tumor Proliferation and Survival in Recipient Cells

GBMs are rapidly proliferating tumors with a tremendous potential to escape cell death. Glioma cells are heterogeneous and are characterized by a highly mutated genome with several tumor promoting genetic and epigenetic modifications. The diversity of transcriptomic profiles observed in glioma cells is mirrored in the EVs derived from these cells. This dynamic EV mediated communication via the transfer of oncogenic proteins, mRNA and miRNA is one of the crucial factors in GBM proliferation and survival (8, 9).

Glioma cells, especially GSCs are vital in supporting other glioma cells to enhance their capability to proliferate and escape cell death. GSCs are a unique subset of glioma cells that reside in perivascular niche (10), and play a key role not only in cell proliferation and survival but also in multilineage differentiation, invasion (11, 12), resistance to chemotherapy (13), and radiation (14) by actively communicating with the surrounding glioma and stromal cells (15). Recent studies have provided a window into the heterogeneity of EVs derived from various subsets of glioma cells and GSCs with respect to their cargo and their function. Spinelli et al. explored EVs released by proneural and mesenchymal GSCs and identified that proneural GSC derived EVs lacked canonical EV markers such as CD9, CD63, and CD81, while they were abundant in mesenchymal GSC derived EVs. They also showed differential uptake of EVs derived from both subtypes by the endothelial cells. This study gives an insight into the heterogeneity of EVs based on glioma cell state and their variable functional effects (16).

Glioma EVs carry the oncogenic epidermal growth factor receptor (EGFR) and its deleted variant III (EGFRvIII) protein and mRNA (5, 17). Importantly, EGFRvIII expressing glioma cells deliver their oncogenic cargo to neighboring naive glioma cells (17). This transfer promotes oncogenic activity and cellular proliferation via activation of transforming pathways such as Akt and MAPK pathways (17). EV mediated delivery of glioma cell derived microRNAs, including miR-451, miR-21, miR-29a, miR-222, miR-30a, miR-92b, miR-221, and miR-23 have been implicated in cell proliferation and inhibition of apoptosis (5, 18, 19). Shi et al. showed that glioma EVs derived from the CSF of patients with recurrent GBM were enriched with miR-21 and the levels of cellular miR-21 affected the cellular and exosomal levels PTEN, RECK, and PDCD4 genes at the protein level and also prevented apoptosis (20). Setti et al. showed that Chloride Intracellular Channel-1 (CLIC1) containing EVs released by GSC stimulate cell growth and proliferation *in vitro* and *in vivo* (21). Annexin A2 has been implicated in driving glioma invasion and progression (22) and has been demonstrated as one of the most abundant proteins in glioma EVs (23), but the functional role of transferring Annexin A2 via EVs in glioma progression has yet to be elucidated. Nevertheless, *in vivo* studies have shown that aberrant expression of miR-1 in glioma cells results in tumor suppression by directly inhibiting Annexin A2 (23). Furthermore, miR-1 is associated with anti-tumor properties in several cancers, including GBMs, and has a potential for targeted therapy (23).

Astrocytes are endogenous native cells of the brain, phenotypically similar to glioma cells and communicate with glioma cells to play major role in promoting tumor growth and survival. They can be transformed to glioma cells *in vitro* and *in vivo* by numerous oncogenes including EGFRvIII, MYC, RAS. Recruitment of reactive tumor associated astrocytes is another possible mechanism of inevitable recurrence of GBM. Studies have shown that radiation and resection associated injury can further cause alterations in the astrocyte's transcriptome and secretome, potentiating tumor aggressiveness. Recent studies also explored EV mediated intercellular communications between glioma cells and astrocytes EVs, and their role in tumor proliferation. Hallal et al. showed that astrocytes take up glioma EVs and undergo phenotypic changes. Glioma EVs induce inhibition of TP53 and activation of MYC signaling pathway in astrocytes leading to a development of pro-inflammatory, tumor-promoting, senescence-associated phenotypes which may promote and support tumor progression (24). Oushy et al. showed that glioma EV-treated astrocytes are shown to have an increased migratory capacity and cytokine production that also promotes tumor growth and proliferation. Astrocytes exposed to glioma EVs also acquired tumor like signaling pathways and exhibited colony forming behaviors suggesting delivery of oncogenic cargo via EVs and may even drive astrocytes to a tumorigenic phenotype (25). The study also showed that glioma EVs modulate surrounding astrocyte signaling to promote evasion of apoptosis by inactivating Bcl-2 associated death promoter (BAD), a pro-apoptotic member of Bcl2 (25). Transfer of miR10b, miR-21, miR-26 from glioma cells to astrocytes also could facilitate their malignant transformation (26–28).

Recently, an elegant study by Abels et al. showed *in vivo* delivery of tumor miRNA cargo to neighboring host microglia cells creating a tumor supporting microenvironment. Specifically, the authors used GFP expressing glioma cells which were implanted into miR-21 *null* mice and upon tumor growth, intracranial cells were sorted based on microglia markers as well as levels of GFP. EV-GFP^pos^ microglia were shown to have a significant reduction in mRNAs, including Btg2, Pdcd4, and Nfat5, direct targets of miR-21. Furthermore, downregulation of Btg2 in microglia led to increased cell proliferation, which suggests that tumor cells deliver specific cargo to regulate their TME to create a more favorable microenvironment for glioma tumor progression (29). Li et al. showed that EVs released by tumor associated endothelial cells contain CD9, which increases GSC proliferation by activating BMX/STAT3 signaling pathways (30). Deng et al. showed that human marrow stromal cells secrete miR-375 containing exosomes which act on glioma cells and inhibit cell progression through SLC31A1 suppression and could be a potential therapeutic target (31). Interestingly, oligodendrocytes inhibit GBM growth and proliferation via WNT inhibitory factor 1 signaling (32) although the exact role of EVs in glioma-oligodendrocyte communication remains unknown. The nature of interactions between glioma cells and endothelial cells, immune cells, and their contributions to tumor proliferation is discussed extensively in the subsequent sections.

## Glioma EVs Induce Angiogenesis

**Figure 2:**
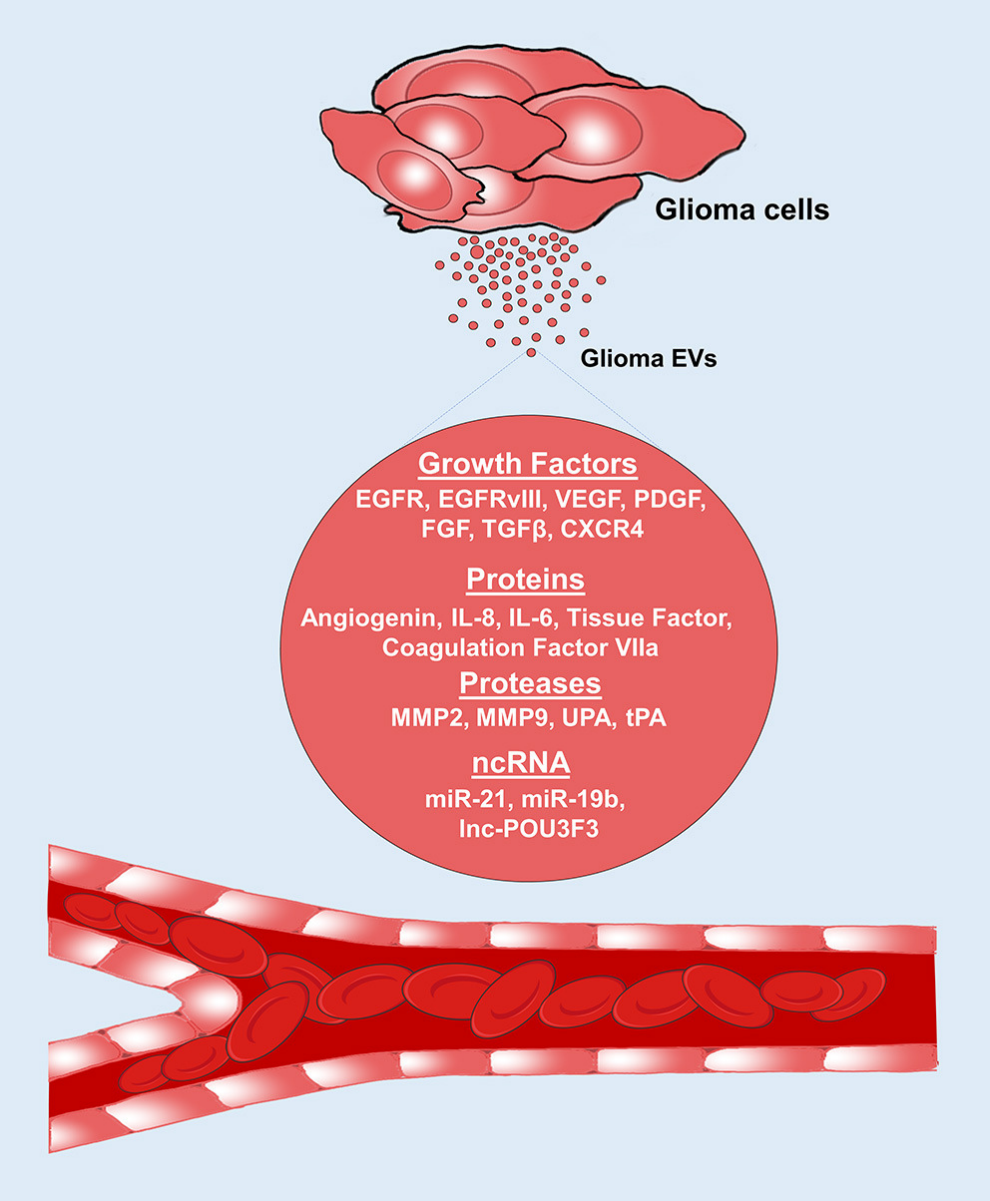
Glioma EVs promote angiogenesis. Glioma EVs contain several proangiogenic factors which induce angiogenesis. EGFR, epidermal growth factor receptor; VEGF, vascular endothelial growth factor; PDGF, platelet derived growth factor, FGF, fibroblast growth factor; TGF-β, Transforming growth factor-β; IL-8, interleukin-8; IL-6, interleukin-6; CXCR4, CXC chemokine receptor type 4; MMP2, matrix metalloproteinase 2; MMP9, matrix metalloproteinase 9; UPA, urokinase type-plasminogen activator; tPA, tissue type-plasminogen activator.

Rapidly growing glioma cells require a constant supply of nutrients and oxygen. Limited blood supply in tumor niche creates an environment which is both hypoxic and nutrient deprived. Angiogenesis allows tumor cells to procure nutrients and oxygen to enhance growth and infiltration (33). Tumor cells support themselves by releasing a multitude of growth factors, soluble factors, and EVs to constantly stimulate angiogenesis to supply this demand. In addition to angiogenesis, mechanisms such as vessel co-option and vascular mimicry also contribute to procuring blood supply (34). Angiogenic factors such as vascular endothelial growth factor (VEGF), fibroblast growth factor (FGF), cytokines, miRNAs, and proteases have pivotal role in angiogenesis (35). Recent studies in leukemia, melanoma, ovarian cancer, and GBM have further uncovered the role of EVs in promoting angiogenesis (5, 36–38). Glioma EVs have been shown to contain various pro-angiogenic factors which dynamically reprogram endothelial cells to stimulate proliferation, migration, differentiation and eventually induce them to organize into new tubular structures to enable sustained growth and proliferation and contribute to angiogenesis (39) (Figure 2).

Several EV mediated angiogenesis drivers have been uncovered. Specifically, glioma EVs containing EGFRvIII protein stimulates VEGF promoter activity and increased VEGF release from glioma cells, enhancing angiogenesis (17). EVs derived from plasma and cerebrospinal fluid (CSF) of GBM patients have been demonstrated to enhance endothelial migration and proliferation of endothelial cells by activating the AKT/beta-catenin pathway shedding light into the functional role of proangiogenic EVs (40). Wide array of pro-angiogenic factors such as VEGF, platelet derived growth factor (PDGF) (33), FGF, angiogenin (5), interleukin-8 (IL-8) (5), IL-6 (5), coagulation factor VIIa (41), tissue factor (33), transforming growth factor beta (TGF-β) (42), CXC chemokine receptor type 4 (CXCR4) (42) and proteases such as matrix metalloproteinases (MMP2), MMP9, urokinase type-plasminogen activator (UPA), and tissue type-plasminogen activator (tPA) (42) have been shown to contribute to angiogenesis. MiRNAs such as miR-21 via VEGF signaling pathway (43) and miR-19b by repressing anti-angiogenic proteins such as thrombospondin-1 and connective tissue growth factor (44) also promote angiogenesis. In contrast, miR-1 has suppressive effects on angiogenesis and its expression is downregulated in patients with GBM (23). More recently, transfer of long intergenic non-coding RNA, linc-POU3F3 via glioma EVs resulted in increased expression of VEGF, FGF, FGFR in the endothelial cells, and promoted migration, proliferation, tube formation *in vitro* and arteriole formation *in vivo* (45). Similarly, another non-coding RNA, linc-CCAT2 derived from glioma EVs promoted angiogenesis *in vitro* and *in vivo* in addition to enhancing Bcl-2 expression and inhibiting Bax and caspase-3 expression in endothelial cells, decreasing apoptosis (46).

Recent single cell genome sequencing analyses have demonstrated a distinct intratumoral heterogeneity for profiles of hypoxia. Each individual tumor demonstrated regions of cells that respond to a range of oxygen tensions (47, 48). Researchers exploring hypoxia mediated GBM driving pathways are uncovering multilevel modulation of genomic as well as secretome profiles to enhance proliferation and survival. Hypoxia triggers glioma cells to release EVs with distinct functional proangiogenic cargo including cytokines, growth factors, proteases, and miRNA to influence endothelial cells to promote angiogenesis (33, 49). Furthermore, these endothelial cells reprogrammed by glioma EVs also secrete potent growth factors and cytokines which stimulate the proliferation of pericytes (via PI3K/AKT signaling), vascular smooth muscle cells, as well as the migration and proliferation of glioma cells (33). Kucharzewska et al. demonstrated pro-angiogenic factors in EVs derived from the plasma of patients with GBM and suggested the possibility that GBM EV molecular signature consisting of proangiogenic mediators such as caveolin 1(CAV1), IL8, PDGFs, and MMPs, that could provide a non-invasive, biomarker profile that reflects oxygenation status and aggressiveness of GBM (33). Hypoxia stimulated glioma EVs promote tumor vascularization, pericyte vessel coverage, cell proliferation, and eventually reduce tumor hypoxia in GBM TME (33, 49). Surveyal of the myriad proangiogenic molecules within glioma EVs provides an insight into the tremendous capabilities of EVs in modulating their cargo to influence the TME to drive angiogenesis to allow survival and proliferation. More *in vivo* functional studies are required to decipher the exact role of tumor derived EV mediated pro-angiogenic pathways which can be actionable by targeted therapies. Interestingly, EVs derived from macrophages, microglia, astrocytes, endothelial cells were shown to release pro-angiogenic cargo in several cancers but their exact contribution in GBM angiogenesis is still unclear (7, 50–53).

Another interesting yet unexplored aspect is the effect of GBM derived EVs on modulating the integrity of blood brain barrier. Additionally, GBM cells releasing high levels of cytokines, acute phase proteins, coagulation factors, and tissue factors cause thromboembolic events in patients with GBM. Recently, some of these factors, specifically tissue factor/VII-a have been demonstrated in glioma EVs. Their exact role in the context of EVs in activating thromboembolic events is still unclear but is a potential area of therapeutic intervention (54, 55). It is logical to think that a stable structure of EVs are effective means of transport of pro-coagulant factors to distant sites to initiate thromboembolic events but further studies are required to demonstrate this phenomenon.

In conclusion, activated angiogenesis cascade in GBM cells produces pro-angiogenic EVs along with a multitude of other angiogenesis promoting changes to fine tune the surrounding environment to make it favorable to sustain tumor growth by stimulating endothelial cells to promote angiogenesis. *In vivo* studies exploring the exact causal effect of glioma EVs on angiogenesis are required to further decipher the exact pathways to identify potential targets for therapy. Although several anti-angiogenic therapies failed to show a survival benefit in randomized controlled trials of patients with GBM, angiogenesis pathways continue to be candidates for newer antiangiogenesis therapies for these highly angiogenic tumors. Currently only bevacizumab, an anti-VEGF showed some improvement in survival in patients with recurrent GBM (56).

## Glioma EV Mediated Reprogramming of Metabolic Activity

Solid tumors such as GBMs are subjected to enormous microenvironmental shifts, which result in genetic, epigenetic, post-transcriptional, and metabolic changes. Reprogramming the metabolic profiles is crucial for the survival of these rapidly growing tumor cells (57). Unlike normal cells, which depend on glucose homeostasis reciprocally controlled by catalytic/oxidative phosphorylation and anaerobic gluconeogenesis pathways, tumor cells rely on glycolysis, pentose phosphate pathway, and alternate sources of energy such as lactate and acetate for energy requirement (58, 59). High levels of lactic acid produced as a result of glycolytic pathway in cancer cells are exported out in response to intracellular pH regulators, providing an alkaline intracellular environment favoring glycolysis. This acidic burden results in toxicity prompting the emergence of invasive cells. Lactate can be re-imported and used as a source of energy (60). The metabolic contents of EVs also favor glycolytic pathways suggesting tumor derived EVs as potential vehicles for outsourcing energy requirements (57, 61). Interestingly, cancer associated fibroblasts were shown to release EVs with substrates including amino acids, lipids, and TCA intermediates that inhibit oxidative phosphorylation and enhance glycolysis. These fibroblast EVs, upon uptake by tumor cells promote glycolytic pathways (62). Additionally, Ronquist et al. showed that EVs released by prostate cancer cells can produce extracellular ATP via glycolysis and also showed reduced ATPase activity when compared to EVs released by normal prostate cells. They showed similar observations in glioma cells compared to normal glial cells. They also demonstrated an energy dependent uptake of EVs in normal and prostate cancer cells. This generated ATP acts as a substrate for surface membrane phosphorylation reactions to promote EV internalization (61). Thus, making EV internalization an energetically favorable event for the recipient cell.

Tricarboxylic acid (TCA) pathway enzyme, isocitrate dehydrogenase (IDH) mutations are implicated in 5% of primary GBMs and 80% of secondary GBMs, and are also associated with the production of an oncogenic metabolite alpha-ketoglutarate (58, 59, 63). Khurshed et al. showed that IDH1 wildtype glioma cells depend on glycolysis and lactate metabolism while IDH1 mutant glioma cells use oxidative TCA pathway (64). IDH-1 mutant transcripts have been found in EVs from glioma tumor cells (65) suggesting that EVs could be involved in promoting neighboring glioma cells to increasingly utilize oxidative pathway for energy production. Oncogenic mutations were also shown to modulate metabolism to support gliomagenesis. Tumor suppressor gene p53 triggers glycolysis, and loss of PTEN activates Akt pathway which stimulates glucose transporter 4 (GLUT 4) dependent glucose uptake (66). c-MYC activation induces glycolysis which facilitates lactate production (67) and mTOR pathway drives anabolic metabolism (58, 59, 68). All these oncogenic proteins have been reported in tumor EVs (69) and could be influencing these metabolic pathways in the recipient neighboring glioma cells to favor progression. Recent studies showed that large oncosomes/ large EVs preferentially contain protein cargo targeted to mitochondrial metabolic processes including VDAC1/2, the solute carriers SLC25A6 and SLC25A5 that are mitochondrial ADP/ATP translocators as well as the ATP synthase subunit ATP5B. Small EV cargo on the other hand contained higher amounts of proteins clustered toward glucose and glutamine metabolism and gluconeogenesis (70). Although both small and large EV subfractions are shown to contain tumor derived cargo useful for tumor biomarking, they subpopulations could be mediating separate wings of metabolic and other functional reprogramming (71). In summary, studies from several cancers hint that tumor EVs drive glycolysis and other tumor supporting energy generating pathways and thus potentiate tumor growth by metabolic remodeling (72) but the exact contribution of EVs in these phenomenon is still unclear. Understanding the spatiotemporal sequence of metabolic alterations and the role of EVs in these aspects can provide insights into the tumor biology as well as offer potential therapeutic targets.

## Glioma EV Mediated Immunomodulation of TME

**Figure 3:**
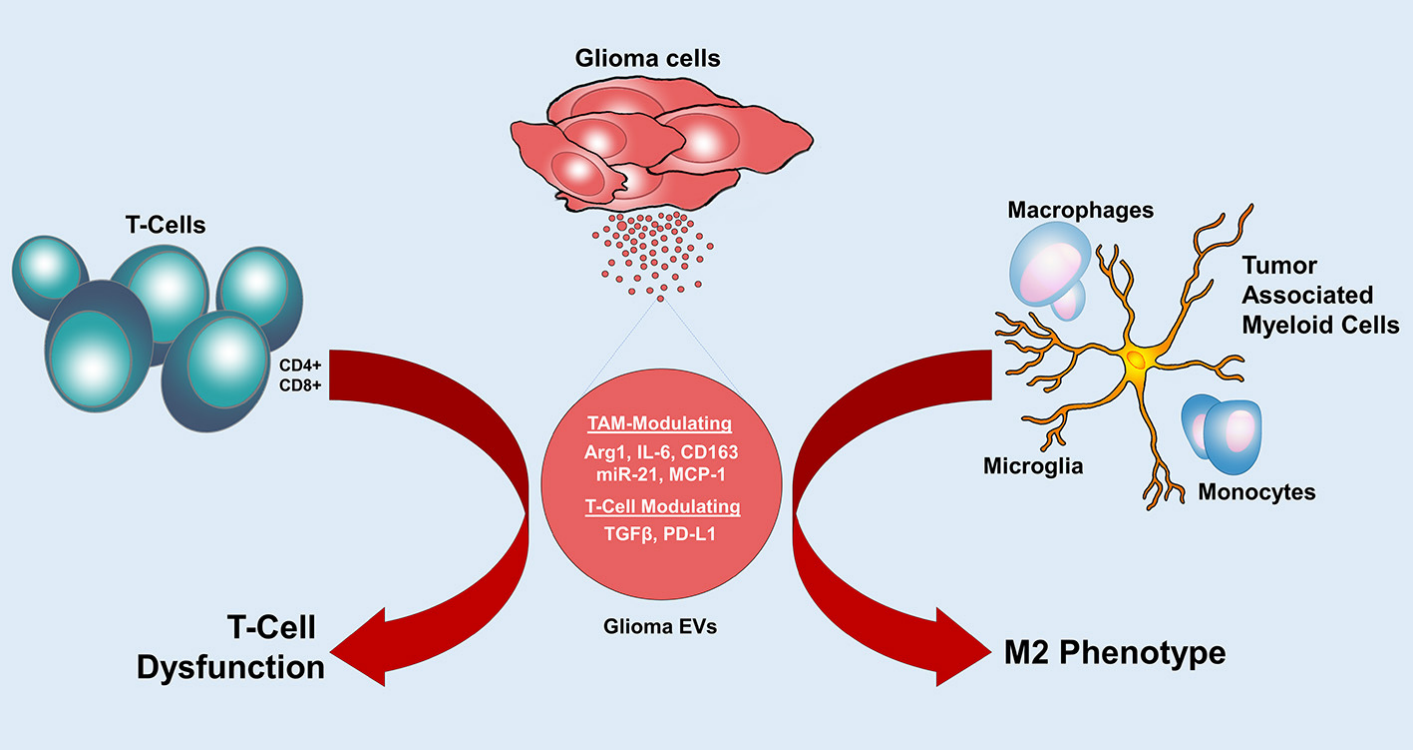
Glioma EVs mediate immunomodulation. Glioma EVs induce an M2 phenotype in tumor associated myeloid cells and cause T cell dysfunction. Arg1, arginase-1; IL-6, interleukin-6; MCP-1, monocyte chemoattractant protein 1; TGF-β, Transforming growth factor-β; PD-L1, programmed cell death 1 ligand.

Normally, in TME natural killer cells (NK cells) and γδT cells provide the first line of defense against tumor cells by direct cytotoxic effects and secretion of high levels of interferon-γ. M1-Macrophages also contribute by exhibiting high levels of phagocytosis and secretion of proinflammatory cytokines. Antigen presenting cells like dendritic cells activate CD4+ and CD8+ T cells to prime T cell activation. CD8+ T cells exhibit direct cytotoxicity to eliminate tumor cells. Overtime, this antitumor response is blunted and altered by glioma cells to create a tumor favoring response. There is NK cell and CD8+ T cell exhaustion, recruitment and expansion of immunosuppressive immune cells, presence of high levels of immunosuppressive factors including cytokines, EVs and a shift in polarization toward a type II immune response with tumor supporting Th2-like cytokine secretion, M2 macrophage polarization, type II NK cells (3). All these factors lead to the immunosuppression exhibited by GBM (73, 74). EVs released by tumor cells within the tumor microenvironment also contribute to lowering immunosurveillance and promoting anti-tumor effector functions, which can in turn drive tumor growth (73, 74) (Figure 3).

Tumor associated myeloid cells include microglia, monocytes and macrophages constitute around 30–50% of the GBM tumor mass (3). Glioma cells release a multitude of factors to actively recruit these myeloid cells to their vicinity (75) and then activate them through their secretomes, including EVs (53). Once activated, tumor associated myeloid cells contribute to tumor proliferation by secreting oncogenic factors such as EGFR (76), extracellular matrix (ECM) remodeling by releasing proteases such as MMP2 and MMP14 and induction of angiogenesis by secretion of proangiogenic factors (53, 77). Glioma cells modulate tumor associated myeloid cells to drive an immunosuppressed state by affecting their ability to activate the immune system (78). *In vitro* studies showed that glioma EV treated monocytes and microglia developed a tumor promoting M2 phenotype. There was upregulation of tumor supporting cytokines including IL-6 while immunogenic cytokines such as IL-16 were downregulated. Recent study demonstrated the functional effects of miR-21 containing glioma EVs in microglial M2 phenotypic transition *in vivo* (79)*. In vitro* M2-phenotypic polarization of peripheral blood monocytes when treated with glioma EVs gives an insight into phenotypic modifications occurring in the myeloid cells in the TME to promote tumor growth (53). Glioma EVs were also shown to suppress the activity of natural killer cells and increase the activity of myeloid derived suppressor cells (MDSC) (80). Ridder et al. showed transfer of Cre mRNA from glioma EVs to MDSC enhanced their activity and produced an immunosuppressive phenotype and miRNA profiles (81). In contrast, EVs derived from some antigen presenting cells containing MHC complexes were shown to directly or indirectly activate CD4+ and CD8+ T cells. Dendritic cell derived EVs induce proinflammatory cytokine profile. Dynamic interplay of pro-tumor and anti-tumor EV mediated immunomodulation exists in the TME, but the eventual tilt in the balance is toward tumor promoting and supporting immune response.

Adaptive immune cells (T cells) are capable of exerting antitumor effects (3). Glioma cells can impair their antitumor function via Fas antigen ligand (FasL), programmed cell death 1 ligand (PDL-1), VEGFA, and EVs (82–84). Glioma EVs containing TGF β suppress T cell activation and IL-2 dependent T cell survival. They also attenuate the ability of CD8+ T cells to express granzymes and IFN γ, reducing their functionality (85). High concentrations of glioma EVs were demonstrated to induce an immunosuppressive phenotype in T cells isolated from peripheral blood (86). Glioma EVs derived from serum of patients with GBM are also associated with cytokines that drive a tumor supporting Th2 phenotype as opposed to Th1 phenotype. GSCs were shown to release EVs containing functional PD-L1, which directly interacts with T cells to suppress their activity. It has also been shown that PD-L1 DNA detected in the plasma of GBM patients correlated with tumor volume and can be used as a biomarker (87). In addition to the transfer of genetic cargo, surface expression of immunosuppressive molecules such as PDL1, FasL, TNF-α, and other decoy ligands for NK cell and CD8+ T cells reduce immune cell recognition by these cells (87). Incubation of EVs isolated from plasma of GBM patients with peripheral blood monocytes from healthy donors led to an inhibitory effect on the proliferation of CD4+ T cells and when incubated with peripheral blood derived monocytes, displayed increased IL-10, arginase-1 production, and downregulation of HLA-DR displaying a phenotype resembling MDSCs. They suggested that glioma EVs suppress T-cell immune response, both directly and indirectly by acting on monocyte maturation. Although the exact role of tumor associated myeloid cells in T cell dysfunction is unknown, glioma EVs may modify myeloid cells to secrete factors that suppress T cell activation (88).

Other immune cells such as neutrophils and mast cells are also recruited into the GBM TME. Tumor associated neutrophils release cytokines, S100A proteins, and elastases to recruit monocytes and more neutrophils. They provide a tumor supporting environment by recruiting myeloid cells in the TME (89, 90). Glioma cells also recruit mast cells which secrete several soluble factors and proangiogenic factors to promote tumor growth and angiogenesis (91). Neutrophils and mast cells have a limited direct role in immunomodulation but help drive tumor promoting mechanisms within the TME. The exact effect of glioma EVs on neutrophils and mast cells is still unclear (3).

These studies highlight the role of EVs in tumor immune escape. Tumor associated myeloid cells and T lymphocytes offer actionable targets to promote anti-tumor immune response. Immune checkpoint inhibitors have been explored as possible anti-tumor immune therapies but with limited success in GBM. Better *in vivo* models are required to study the effects of EVs in the dynamic immune microenvironment (2). Further research could unfold pathways that can be targeted to enhance an antitumor immune response by overcoming the immunosuppressive signals delivered by EVs.

## EVs Play a Role in Acquiring Drug Resistance

**Figure 4:**
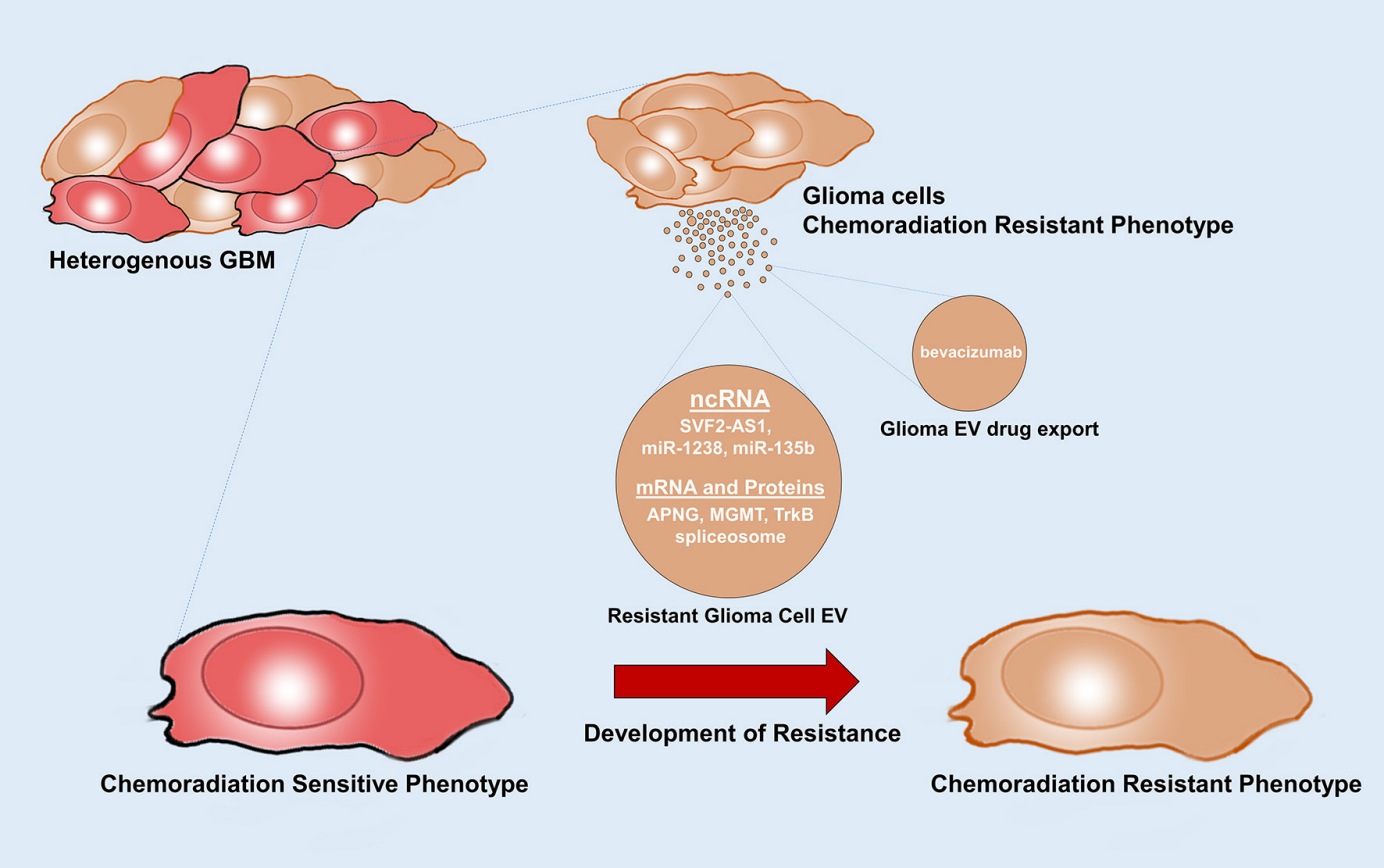
Glioma EVs mediate therapy resistance. Glioma EVs mediate the transfer of factors that induce a chemoradiation resistance phenotype in chemoradiation sensitive glioma cells. Glioma EVs also mediate drug export. APNG, alkyl purine-DNA-N-glycosylase; MGMT, O(6)-methylguanine DNA methyltransferase.

Treatment failure and tumor recurrences are almost always inevitable in patients with GBM. Several factors have been implicated in this elusive ability of GBM cells to develop resistance to chemoradiation and targeted therapy. Modalities that glioma cells use to develop resistance to therapy include heterogeneity of glioma cells, phenotypic modulation, and reacquisition of stemness in GSCs when exposed to chemoradiation and other therapies, acquisition of salvage pathways, drug metabolism alterations, enhanced DNA repair, upregulation of drug efflux, and inactivating pathways (92). Recent studies uncovered the role of EVs in acquiring drug resistant phenotypes (39). Cancer cells dynamically modulate the composition of their EV cargo in response to therapy, as one additional way to acquire capabilities to resist the therapy and proliferate. Multiple EV mediated mechanisms such as transfer of drug efflux pumps, functional mRNAs, miRNAs, long non-coding RNAs, spliceosomes, fusions, and other resistance acquiring products, reduced drug uptake, intracellular drug inactivation and repair of drug induced DNA damage or defects in DNA response pathway contribute to EV orchestrated drug resistance (93) (Figure 4). Hypoxia in GBM induces EV signaling which is also shown to contribute to chemoresistance (33, 94).

GSCs are the main drivers of resistance to therapy. They accumulate novel mutations and further generate heterogenous resistant clones of glioma cells. They orchestrate the transfer of resistance acquiring products from resistant cells to sensitive cells (95). A multitude of RNAs including functional mRNAs, miRNAs, long non-coding RNAs carried by EVs induce drug resistance in sensitive cells. Levels of the DNA repair enzymes alkyl purine-DNA-N-glycosylase (APNG) and O(6)-methylguanine DNA methyltransferase (MGMT) are inversely correlated to response to the gold standard chemotherapeutic temozolomide(TMZ) (96). Shao et al. showed that EVs containing MGMT mRNA have been demonstrated to accurately reflect the levels of these enzymes in parental cells and in patients throughout treatment and therefore could serve as a potential biomarker of chemotherapy response during drug treatment. The transfer of APNG and MGMT mRNAs, could generate these enzymes in the recipient cells and help repair and reverse the damage to DNA caused by TMZ (19). Interestingly, EVs released by tumor associated astrocytes were also shown to contain MGMT mRNA which induces TMZ resistant phenotype in sensitive glioma cells (97). High levels of miR-1238 were detected in EVs isolated from TMZ resistant cells as well as plasma of GBM patients. Transfer of glioma EV derived miR-1238 induced TMZ resistance in non-resistant glioma cells. A loss of miR-1238 may sensitize resistant glioma cells by targeting CAV1/EGFR pathway and could be a potential therapeutic target (98). Zhang et al. showed that exosomal transfer of long non-coding RNA SBF2-AS1 enhances chemoresistance to TMZ in glioma cells (99). Studies in several cancers have shown that EV mediated transfer of drug efflux pumps such as P-glycoprotein, MRP1, ABCG2, and ABCA3 induced resistance in recipient cells (100–103).

Additionally, recent studies also highlight the role of TrkB, a member of the neurotrophin tyrosine kinase receptor-1 family, which is highly expressed in EVs from GSCs. Glioma EV mediated transfer of TrkB is shown to induce therapeutic resistance when taken up by non-therapy resistant cells. TrkB was also detected in plasma of GBM patients and its level correlates with tumor progression and aggressiveness (104). Pavlyukov et al. showed that apoptotic glioma cells release spliceosomes which imparts therapy resistance and aggressive migratory phenotype (105). Zeng et al. showed that glioma cells harboring pro-oncogenic fusion, PTPRZ1-MET fusion (ZM fusion), release EVs that impart TMZ resistance to non-ZM fusion cells. They also induce epithelial to mesenchymal transition and promote migration and invasion (106). EVs produced by glioma cells following bevacizumab treatment directly captured the drug during its release and promoted resistance to bevacizumab. Interestingly, inhibition of glioma EVs improved antitumor effects of bevacizumab (107).

EV mediation acquisition of radioresistance has also been demonstrated in recent studies. Xiao et al. showed that glioma EV derived miR-135b is implicated in transfer of resistance to radiation to other radiosensitive glioma cells by activating miR-135b-GSK3β pathway (106, 108). Glioma EV mediated transfer of HIF-1α promotes radioresistance in sensitive glioma cells.

Although, these few examples seem quite promising it remains widely unexplored and elusive whether EVs are indeed significant contributors to either intrinsic or acquired resistance. Monitoring tumor derived EV profile could provide real-time insights into the altering GBM phenotype in response to therapy. This can allow us to effectively strategize therapeutic options to tackle this elusive tumor. EV-based therapeutics utilizing functional delivery of specific RNAs via EVs as therapeutic delivery systems to alter the phenotype of malignant cells could prove an attractive prospect. Technology is now emerging allowing targeted use of extrinsically generated EVs in order to counteract tumors.

## Tumor EVs Promote Glioma Cell Migration and Invasion

GBMs infiltrate rapidly infiltrating by generating satellite tumors making complete surgical resection impossible eventually resulting in recurrence (109). The stemness phenotype of GSCs, glutamate induced Ca 2+ influx (110), Wnt (111, 112), and PI3/Akt (113) signaling pathway induced MMP release, β catenin degradation pathway (114) as well as the release of soluble factors, proteases, glycosidases allow GBM cells to invade and migrate locally (115). Glioma EVs also contribute to this phenomenon. Glioma EVs are shown to impart migratory phenotypes in the neighboring glioma cells to promote invasion (105). Hypoxic environments stimulate glioma cells to secrete EVs that contain proteins involved in the actin cytoskeleton regulation, extracellular matrix-receptor interactions, focal adhesion and leukocyte trans-endothelial migration suggesting that hypoxic glioma derived EVs promote a migratory phenotype in glioma cells (33, 53). Glioma EV mediated transfer of HIF-1α promotes invasive capacity along with inducing radioresistance in sensitive glioma cells (116). Glioma EVs containing immunoglobulin superfamily protein L1CAM were shown to promote cell motility, proliferation and invasiveness in glioma cells *in vitro* (117).

Mesenchymal subtype of GBM is the most aggressive subtype and EVs derived from mesenchymal GBM cells were demonstrated to affect the surrounding cells in the TME contributing to cell invasion (118, 119). MiR-5096 has been recently demonstrated to increase the outgrowth of filopodia and the invasive ability of glioma cells (120). MiR-218 (121), miR-101 (122), miR-152 (123), and miR-149 (124), all involved in invasion and migration of glioma cells, are downregulated in GBMs, ultimately contributing to increased glioma invasiveness. Semaphorin 3A found in blood and CSF derived EVs from GBM patients promotes vascular permeability by disrupting endothelial barrier integrity (125). Vascular integrity can also be modulated remotely by delivery of miR-132 in EVs released from neurons, via the indirect upregulation of the adherens junction protein, VE-cadherin (126). The exact role of EVs in glioma cell migration, invasion, and remodeling ECM is still under active investigation and this offers potential actionable targets to inhibit GBM progression and invasiveness.

## Conclusion

Translational interest in the EV space has focused on utilizing EV based biomarkers for cancer diagnosis and monitoring. Considering the multifaceted role of EVs derived from glioma cells and stromal cells in modulating TME, more studies are warranted to further clarify their implications in tumor growth and evolution. Lack of proper *in vivo* models, *in vivo* EV analysis tools and inability to isolate and study EV subtypes have hampered researchers to study the specific functional implications of EV subtypes in various hallmarks of cancer. This comprehensive review focused on the multidimensional role of EVs in tumor proliferation, reprogramming metabolic activity, inducing angiogenesis, escaping immune surveillance, acquiring drug resistance, migration, and invasion highlights the current status of understanding of the role of GBM in TME. Further research can provide novel therapeutic targets to effectively fight this deadly tumor. Ability to longitudinally study and characterize EVs in the peripheral circulation in patients with GBM can provide real time insights into the dynamic alterations in the landscape of the tumor and its microenvironment to design strategies to counter the rapidly evolving tumor growth and proliferation.

## Author Contributions

LB and BC conceptualized and supervised the review. AnuroopY and AnudeepY contributed equally to drafting the manuscript. KM, AnuroopY, and AnudeepY prepared the figures. AnuroopY, AnudeepY, KM, KK, LB, and BC reviewed and edited the manuscript.

### Conflict of Interest

The authors declare that the research was conducted in the absence of any commercial or financial relationships that could be construed as a potential conflict of interest.

## Abbreviations

GBM: glioblastoma
TME: tumor microenvironment
EVs: extracellular vesicles
GSC: glioblastoma stem cells
EGFR: epidermal growth factor receptor
VEGF: vascular endothelial growth factor
PDGF: platelet derived growth factor
FGF: fibroblast growth factor
TGF-β: Transforming growth factor-β
IL-8: interleukin-8
IL-6: interleukin-6
CXCR4: CXC chemokine receptor type 4
MMP2: matrix metalloproteinase 2
MMP9: matrix metalloproteinase 9
UPA: urokinase type-plasminogen activator
tPA: tissue type-plasminogen activator
CLIC1: Chloride intracellular channel-1
APNG: alkyl purine-DNA-N-glycosylase
MGMT: O(6)-methylguanine DNA methyltransferase.
